# Recurrent Laryngeal Nerve Intraoperative Neuromonitoring Indications in Non-Thyroid and Non-Parathyroid Surgery

**DOI:** 10.3390/jcm13082221

**Published:** 2024-04-11

**Authors:** Aina Brunet, Aleix Rovira, Miquel Quer, Alvaro Sanabria, Orlando Guntinas-Lichius, Mark Zafereo, Dana M. Hartl, Andrés Coca-Pelaz, Ashok R. Shaha, Jean-Paul Marie, Vincent Vander Poorten, Cesare Piazza, Luiz P. Kowalski, Gregory W. Randolph, Jatin P. Shah, Alessandra Rinaldo, Ricard Simo

**Affiliations:** 1 Department of Otorhinolaryngology, Head and Neck Surgery, Hospital Universitari Bellvitge, Universitat de Barcelona, 08907 Barcelona, Spain; 2 Institut d’Investigació Biomèdica de Bellvitge, L’Hospitalet de Llobregat, 08908 Barcelona, Spain; 3 Department of Otorhinolaryngology, Head and Neck Surgery, Guy’s and St Thomas’ NHS Foundation Trust, London SE1 9RT, UKricard.simo@gstt.nhs.uk (R.S.); 4 Department of Otorhinolaryngology, Head and Neck Surgery, Hospital de la Santa Creu i Sant Pau, Universitat Autònoma de Barcelona, 08025 Barcelona, Spain; 5 Department of Surgery, Universidad de Antioquia, Hospital Universitario San Vicente Fundación, CEXCA Centro de Excelencia en Enfermedades de Cabeza y Cuello, Medellin 1226, Colombia; 6 Department of Otorhinolaryngology, Head and Neck Surgery, Jena University Hospital, 07747 Jena, Germany; 7 Department of Head and Neck Surgery, MD Anderson Cancer Center, Houston, TX 77030, USA; 8 Thyroid Surgery Unit, Department of Otorhinolaryngology Head and Neck Surgery, Institute Gustave Roussy, 94805 Paris, France; dana.hartl@gustaveroussy.fr; 9 Department of Otolaryngology, Hospital Universitario Central de Asturias, University of Oviedo, ISPA, IUOPA, CIBERONC, 33011 Oviedo, Spain; 10 Department of Head and Neck Surgery, Memorial Sloan-Kettering Cancer Center, Medical College, Cornell University, New York, NY 10065, USA; 11 Department of Otorhinolaryngology Head and Neck Surgery, Institute of Biomedical Research, University Hospital Rouen, 76000 Rouen, France; jean-paul.marie@chu-rouen.fr; 12 Department of Otorhinolaryngology Head and Neck Surgery, University Hospitals Leuven, 3000 Leuven, Belgium; 13 Department of Otorhinolaryngology Head and Neck Surgery, ASST Spedali Civili of Brescha, School of Medicine, University of Brescia, 25123 Brescia, Italy; 14 Department of Otorhinolaryngology Head and Neck Surgery, A.C. Camargo Cancer Center, Faculty of Medicine, University of Sao Paulo, São Paulo 03828-000, Brazil; lp_kowalski@uol.com.br; 15 Department of Otorhinolaryngology, Division of Thyroid and Parathyroid Endocrine Surgery, Massachusetts Eye and Ear Infirmary, Boston, MA 02114, USA; 16 Department of Surgery, Memorial Sloan-Kettering Cancer Center, Weil Medical College, Cornell University, New York, NY 10065, USA; 17 School of Medicine, University of Udine, 33100 Udine, Italy; 18 King’s College London, London SE5 8AF, UK

**Keywords:** recurrent laryngeal nerve, intraoperative nerve monitoring, recurrent laryngeal nerve palsy, oesophagus, trachea

## Abstract

Introperative nerve monitoring (IONM) of the recurrent laryngeal nerve (RLN) is a well-established technique to aid in thyroid/parathyroid surgery. However, there is little evidence to support its use in non-thyroid or non-parathyroid surgery. The aim of this paper was to review the current evidence regarding the use of IONM in non-thyroid/non-parathyroid surgery in the head and neck and thorax. A literature search was performed from their inception up to January 2024, including the term “recurrent laryngeal nerve monitoring”. IONM in non-thyroid/non-parathyroid surgery has mainly been previously described in oesophageal surgery and in tracheal resections. However, there is little published evidence on the role of IONM with other resections in the vicinity of the RLN. Current evidence is low-level for the use of RLN IONM in non-thyroid/non-parathyroid surgery. However, clinicians should consider its use in surgery for pathologies where the RLN is exposed and could be injured.

## 1. Introduction

Over recent years, the paradigm for thyroid and parathyroid surgery has been influenced by the introduction of intraoperative nerve monitoring (IONM). IONM has a 99.6% negative predictive value for the absence of vocal fold paralysis (VFP) and a 75% positive predictive value for VFP when there is an intraoperative loss of signal [1]. Most thyroid-related professional societies and working groups around the world now support the regular use of IONM in thyroid and parathyroid surgeries [1,2,3].

There is plenty of reported evidence on the risk of injury to the recurrent laryngeal nerve (RLN) in cardiac or intrathoracic surgeries and particularly for lung cancer [4] but very little on the use of IONM in these kinds of procedures where the RLN is exposed.

There are a number of surgical procedures in the lower cervical area or the thoracic inlet in which the RLN is exposed, identified, or even mobilised in order to successfully complete the procedure. These procedures would include, amongst others, tracheal resections, high oesophageal resections and anastomosis, pharyngeal pouch surgery, thymectomies, the excision of neurogenic neoplasms, bronchogenic tumours and cysts, and paratracheal and superior mediastinal dissection for melanoma and germinal cell tumours [5]. Nonetheless, there is currently a lack of evidence for the use of IONM in these non-thyroid/non-parathyroid surgeries.

Due to the paucity of evidence and the heterogeneity of the pathology, a systematic review cannot be performed to study this research question. In view of this, our aim was to provide a narrative review of the available evidence on the use or potential use of IONM in non-thyroid/non-parathyroid surgery supplemented with the experience of the authors in their tertiary referral practice and to formulate an evidence-based proposal of its potential utility.

The purpose of this review is to discuss the current evidence on the use of the RLN IONM as defined in the Materials and Methods and to provide awareness and recommendations for its use in non-thyroid and parathyroid surgeries.

## 2. Materials and Methods

A PubMed, Embase, and Cochrane literature search was performed from their inception up to and including January 2024, using the following search term strategy: “recurrent laryngeal nerve monitoring” and “pharyngeal pouch surgery”, “oesophageal surgery”, “esophageal surgery”, “thymus surgery”, “tracheal surgery”, “thoracic inlet surgery”, “video-assisted thoracoscopic surgery”, and “VATS”.

For the purpose of this review, the authors addressed only the RLN in those procedures where the nerve can be exposed, identified, and potentially mobilised. The authors excluded all surgeries involving the vagus nerve; those cardiac or thoracic procedures in which, due to technical limitations, the IONM is not possible; and anterior spinal surgery where the RLN is not routinely exposed, as the approach to the anterior spine involves the application of special self-retainer instruments retracting the thyroid gland. We also agreed to limit the review to adult patients. Cervical spine surgery can also be associated with other neurogenic deficits, which would make the findings very heterogeneous. Studies not in English were excluded from this search.

Each section was divided into three parts: (1) surgical anatomy, approach, and potential nature of injury of the RLN; (2) results and outcomes found in the literature; and (3) current or proposed indications.

No research ethics committee approval was necessary for this study.

## 3. Results

### 3.1. Anatomy of the Recurrent Laryngeal Nerve

The RLN is a branch of the vagus nerve. The right and left RLNs are asymmetrical. The right vagus runs posterior to the superior aspect of the right lung where the RLN is originated and loops around the subclavian artery. The left RLN originates from the left vagus nerve, and it passes below the aortic arch. Their name “recurrent” is due to the fact that the nerves course superiorly in the opposite direction as the vagus nerve, passing posteriorly to each thyroid lobe and lateral to the tracheoesophageal groove, with a slightly diverse course from the right to left side. They enter the larynx posterior to the cricothyroid joint on each side, below the inferior constrictor (or crico-pharyngeus) muscle, becoming the inferior laryngeal nerve [6]. The left RLN creates an angle of between 15 and 30° relative to the trachea, whereas this angle is more open in the case of the right RLN [7].

The RLN innervates all intrinsic muscles of the larynx except the cricothyroid muscle, which is innervated by the external branch of the superior laryngeal nerve. It also has visceral autonomous and sensory branches that innervate the inferior region of the glottis, subglottis, and trachea and branches to the crico-pharyngeus and thyro-pharyngeus muscles preceding its entrance to the larynx [6]. The existence and variability of connections should be noted between the recurrent nerve and the two branches of the superior laryngeal nerve, which give a complex pattern that justifies the variability in the final position of the vocal fold in different patients after similar recurrent nerve injury [8].

It has been recognised that both the RLN and the external branch of the superior laryngeal nerve (EBSLN) are susceptible to injury during thyroid and parathyroid surgery. Nevertheless, they are also at risk of injury in non-endocrine procedures that involves working near the area in proximity to their route in the cervico-thoracic area. These procedures include, amongst others, tracheal resections; upper oesophageal resections; pharyngeal pouch surgery; thymectomies; and the excision of neurogenic neoplasms, bronchogenic tumours, and cysts.

RLN injury, being one of the most devastating complications of thyroid and parathyroid surgery, can cause significant patient morbidity as it will cause VFP or paresis, resulting, in many cases, in dysphonia, dyspnea on exertion, and an increased risk of aspiration [6], which can also result from the other types of surgeries that are the subject of this review.

### 3.2. Causes of Injury

During lower cervical or cervico-thoracic surgery, the RLN is undoubtedly at risk of injury, and it may be a source of postoperative complications as well as medico-legal issues [5]. Mechanisms of injury include compression, vascular ischaemia, stretching, severing, or thermal damage [9]. Thermal injury can occur up to 1 cm from the tip of monopolar or bipolar cautery devices, or other energy devices. Hence, in the absence of IONM, neural injury may not be identified during the surgery [5,10].

### 3.3. Current Indications for IONM of the RLN

The main applications and indications for IONM have extensively been described by multiple authors and international study groups. Also, the benefits of RLN IONM have also been addressed in multiple clinical guidelines such as the American Thyoid Association (ATA) [1,11,12]. IONM aids with identification and dissection of the RLN, the prognostication of postoperative neural function, and lesion site identification [3]. Moreover, its judicious use helps in reducing the most feared complication in this field: unexpected bilateral RLN palsy.

IONM has been shown to have a 99.6% negative predictive value for the absence of VFP in the absence of a loss of signal. Its positive predictive value for postoperative VFP in the presence of a loss of signal is 75% [1].

Dionigi et al. [13] described five advantages in performing IONM during thyroid surgery, which also apply to oesophageal surgery [14]: (1) it facilitates RLN identification, (2) it enables the testing of RLN function, (3) it enables corrective action during surgery during the lymphadenectomy procedure, (4) it enables the evaluation of RLN function by vagus nerve stimulation before proceeding to the contralateral site, and (5) for novice surgeons, it increases confidence in performing these procedures.

IONM is now commonly used in many centres around the world during thyroid and parathyroid surgery. However, the same is not true for non-thyroid or non-parathyroid procedures despite the inherent high risk of injury to the RLN [15].

### 3.4. Types of RLN Monitoring

In 1965, Shedd and Durham used intermittent IONM in thyroid surgery to prevent RLN injury [16]. It has shown to contribute to the intraoperative localisation of the RLN [17]. This technique allows the assessment of unclear structures with a handheld stimulation probe before transection. The suprathreshold stimulation at 1–2 mA of the vagal nerve and RLN is performed using a handheld probe [18]. The International Neural Monitoring Study Group (INMSG) recommends the assessment of the vagal nerve and RLN before and after the surgery [19].

In 2000, Lamade et al. proposed the continuous intraoperative nerve monitoring (CIONM), which allows a real-time evaluation of the outcome of surgical manipulation on RLN function [17,18,20,21]. CIONM helps identify adverse electromyographic changes early on to guide the surgeon in sudden corrective manoeuvres aiming to prevent severe RLN injuries [20,22].

## 4. Discussion

### 4.1. Tracheal Resections

- Surgical anatomy and approach [23]

The RLN runs most commonly (46–55%) deep into the inferior thyroid artery (ITA), superficial to it (10–12%), or between the terminal branches of the ITA (33–35%) [5,24]. Finger-tip palpation gently rolling the area of the nerve, which will feel like a tiny cord, against the trachea could sometimes aid in RLN identification, although this could also cause stretch injury. Nonetheless, this method is not as accurate as visual identification [2,23], which remains the gold standard in thyroid surgery. Visual RLN identification does not mean functional integrity of the nerve that can only be assessed by electrical stimulation [2]. Thyroid isthmusectomy or isthmusotomy along the midline (if thyroidectomy is not needed) and lateralization of the thyroid lobes allow us to address the trachea and protect the RLNs. Moreover, subperichondrial resection of the trachea and judicious use of bipolar cautery close to the RLN entry point allow protection of the nerve. Nonetheless, in revision surgery, the RLN may be intimately adherent to the trachea and the nerve can be at increased risk of injury due to the abundance of scar tissue. Therefore, the dissection plane should be even more strictly along the tracheal wall [25,26].

- Outcomes

The rate of permanent RLN injury after tracheal resection in the literature is 3.1–8.2% [25]. Only two studies were found in our search using the terms “tracheal surgery” AND “recurrent laryngeal nerve monitoring”. The first study was published by Piazza et al. in 2014 which included 137 patients with both benign (63.5%) and malignant (36.5%) tracheal stenosis. It reported a 3.6% rate of RLN palsy in adult tracheal resections without IONM [27].

The second study published by Kadakia et al. in 2017 analysed the use of IONM in tracheal resection for non-thyroid pathology in 110 adult patients. IONM was not used for the 49 patients included in the first 10-year period, and a rate 13.1% of RLN injury was reported, which decreased to 6.6% during the long-term period. It was used for the remaining 61 patients that were included in the second 10-year period, with a rate of RLN paralysis of 14.3%, and the long-term paralysis dropped to 8.2%. No significant differences in nerve function preservation were demonstrated between the two groups, but since the prevalence of RLN injury during this procedure is relatively low, the authors recommended a multicentric study with larger numbers of patients. This is yet to be published according to our literature review [25].

- Potential Indications

In non-thyroid benign pathology (i.e., outside the indications for tracheal resection due to thyroid cancer invasion), the most common causes of tracheal resection are tracheal stenosis (idiopathic or post intubation) or tracheoesophageal fistula [25]. Subperichondrial dissection should be considered in order to protect the RLN without directly identifying it [25]. The two clinical scenarios in which IONM should be considered are (1) revision surgery as well as (2) upper crico-tracheal resections. In the first situation, the RLN is at the greatest risk of injury as it is usually submersed into scar tissue, making its identification and lateralisation after thyroid isthmectomy more difficult [26]. In the second scenario, it is obvious that the RLN can be exposed and transected if the resection is performed too laterally [26].

### 4.2. Oesophageal Surgery

- Surgical anatomy and approach

In upper oesophageal surgery and especially in revision surgery, surgeons most often approach the left tracheoesophageal groove in order to achieve upper oesophageal adequate margin-free resections and anastomoses. In these cases, the RLN is identified, appropriately dissected, and mobilised to avoid injury [9]. Like in revision surgery in thyroid and parathyroid indications, as well as in revision upper oesophageal surgery, the risk of injury is likely to be higher as compared to primary surgery.

- Outcomes

There are several studies analysing the role of IONM in oesophageal surgery. Our search found 26 studies using “oesophageal surgery” AND “recurrent laryngeal nerve monitoring” terms and “esophageal surgery” AND “recurrent laryngeal nerve monitoring” terms [14,15,28,29,30,31,32,33,34,35,36,37,38,39,40,41,42,43,44,45,46,47,48,49,50,51] (Table 1). Five of them corresponded to case reports, which described the IONM or IONM in anatomical variations or atypical cases as right aortic arch or an aberrant right subclavian artery. All of them concluded that IONM may be useful [14,28,29]. There were also three review articles: Garas et al. [15] reviewed 125 papers and they supported the use of IONM during high mediastinal lymph node dissection in three-stage oesophagectomy. Yip et al. [45] described advances of minimally invasive oesophagectomy, including IONM. Wang et al. [42] recently published a systematic review and meta-analysis which concluded that IONM can minimise RLN palsy, although it did not show significant benefit in reducing aspiration or pneumonia. Zhong et al. compared two groups of 54 and 61 patients who underwent oesophageal surgery (with and without IONM, respectively). They concluded that IONM helps in increasing the number of identified and removed metastatic periesophageal lymph nodes and also decreases the incidence of RLN palsy and the related morbidity of postoperative pneumonia [31]. More recently, continuous intraoperative nerve monitoring (CIONM) has been described during thoracoscopic oesophagectomy, which allows the surgeon to anticipate imminent damage to the RLN in order to prevent permanent injury [32]. Nitta et al. recently published a case report on the use of IONM by combined intraoperative identification and monitoring in surgical resection of oesophageal carcinoma and found the technique useful for preventing injury to the nerve [14].

- Potential indications

The incidence of RLN injury and thus the need to use IONM vary according to the increasing extent of lymph node dissection, more complex surgical technique (two or three stages), and increasing size and T category of the primary oesophageal tumour. A high lymph node yield and pT3-pT4 category have been correlated with RLN injury [52]. Moreover, RLN IONM may also have a role during upper mediastinal lymph node dissection for oesophageal cancer [53]. The transhiatal approach including a cervical anastomosis for oesophageal cancer carries the highest incidence of RLN injury and is thus a good indication for the use of IONM [5].

### 4.3. Cervical Thymomas

- Surgical anatomy and approach

Cervical thymomas can be located between the trachea and the common carotid artery [54]. Occasionally, they are in continuity with the inferior pole of the thyroid gland and thus can mimic thyroid gland lesions preoperatively [55,56].

Surgery for cervical thymomas includes a transverse cervical incision, and identification of the RLN is useful, as some of the cases can extend to the upper mediastinum [57].

- Outcomes

Although there are no data published in the literature, IONM may be intuitively useful in surgery for cervical thymomas as they are anatomically located in an area with a close relationship to the thyroid and parathyroid glands.

- Potential indications

IONM should be considered when operating cervical thymomas in close relation to the thyroid gland.

### 4.4. External Approach to Zenker’s Diverticulum for Excision of the Pharyngeal Pouch

- Surgical anatomy and approach

The incidence of RLN injury following open pharyngeal surgeries has been described to be between 2 and 23% [58]. The close proximity of the pharyngeal pouch with the cricopharyngeus muscle puts the RLN at risk of injury during open repair [58].

- Outcomes

Coughlan [58] studied eight patients that underwent pharyngeal pouch surgery and IONM. Among them, seven patients had a Zenker diverticulum (ZD), all over 3 cm in size, and the remaining patient had a Killian–Jamieson diverticulum (KJD). The RLN was identified in two cases. The first one was a patient with a ZD measuring 12 cm and the second case was a KJD. The RLN is at a higher risk of injury during surgery for a KJD as it enters the larynx through the Killian–Jamieson triangle just below the level of the cricopharyngeus muscle, which is the origin of the KJD [59]. In ZD, the expansion of the diverticulum can reach the level of the tracheoesophageal groove, which would put the nerve at risk of injury in its proximal route. Finally, this procedure may actually worsen the symptoms of the primary disease, or even significantly worsen them, if the nerve is injured [58].

- Potential indications

Based on the varied risk of injury to the RLN, IONM should be considered in external surgical approaches to a pharyngeal pouch. It should especially be advised in surgery for large pharyngeal pouches [58], in patients requiring revision surgery, and particularly in those who, during the initial procedure, had complications such as fistula or neck abscess.

### 4.5. Thoracic Inlet Neurogenic Tumours

- Surgical anatomy and approach

Multiple nerves are located in the thoracic inlet region, such as the sympathetic plexus and the lower cranial nerves (phrenic nerve, vagus nerve, and recurrent laryngeal nerve) [60].

- Outcomes

There are no studies to our knowledge analysing the role of IONM in surgery for thoracic inlet neurogenic tumours.

- Potential indications

In the surgical treatment of thoracic inlet neurogenic tumours, IONM should be considered as it would aid in identifying the RLN superiorly and it could be followed downwards, especially in large tumours and in revision scenarios. Careful evaluation with relevant cross-sectional imaging would aid in identifying the potential position and or distortion of the RLN.

### 4.6. Video-Assisted Thoracoscopic Surgery

- Surgical anatomy and approach

As the left RLN is located more caudal and dorsal than the right RLN, it is injured more frequently during lung surgery. This risk is particularly higher for patients requiring upper mediastinal lymph node dissection.

- Outcomes

Only one study described the use of recurrent laryngeal nerve monitoring on video-assisted thoracoscopic surgery (VATS). Chai and Lee recently investigated the feasibility and safety of using continuous intraoperative nerve monitoring (CIONM) during VATS lobectomy for early-stage lung cancer [61].

- Potential indications

IONM could be used in VATS lobectomy and may be helpful in preventing RLN injury during VATS left lobectomy.

### 4.7. Other Pathologies

- Surgical anatomy and approach

Other thoracic inlet pathologies in close proximity to the RLN could include, amongst the others, tracheoesophageal fistula, large dermoid tumours, teratomas, bronchogenic cysts, and left metastatic dissemination along the thoracic duct of seminomas and other uro-genital tumours.

Tracheoesophageal fistula is commonly related to neck infection and, therefore, scar tissue formation. Hence, there is usually marked adherence of the RLN and oesophagus to the trachea. Although in tracheal surgery, one should stay as close as possible to the trachea during dissection [26], in tracheoesophageal fistula repair, one needs to separate the trachea from the oesophagus, putting the RLN at higher risk of injury.

In thoracic inlet pathologies, the patient is placed in the supine position with the head hyperextended. A cervical incision is performed, extending downwards to the upper mediastinum. The neurovascular structures are identified in the neck and followed inferiorly into the upper thorax [62]. The RLN is identified in the tracheoesophageal groove [63,64]. The position of the RLN will vary depending on the position and extent of each individual tumour.

- Outcomes

No studies have described the role of IONM in the above-mentioned diseases.

- Potential indications

If in doubt, surgeons undertaking procedures for these pathologies should consider the use of IONM.

### 4.8. Limitations of This Study

Limitations of this study are the mere availability of a few low-evidence studies including only retrospective case series and case reports assessing the feasibility and role of IONM of the RLN in non-thyroid and non-parathyroid surgeries. This may impact on the validity and generalisability of this study.

Some evidence has been published in favour of the use of IONM of the RLN for oesophageal surgery. One published retrospective study did not find an advantage in the use of IONM for tracheal resection anastomosis, but a prospective methodology and sufficient statistical power are lacking. We found no studies concerning the use of IONM for pharyngeal pouch/diverticulum surgery, cervical thymomas, tracheoesophageal fistulas, or thoracic inlet tumours, even though the relevant anatomy would suggest that IONM may improve postoperative outcomes by facilitating RLN dissection and preservation and by potentially reducing the risk of uni-or bilateral RLN injury.

### 4.9. Value of This Study

Up until now, no studies have summarised the potential utility of the RLN IONM in non-thyroid and non-parathyroid surgery. This is the first review of this topic in the English literature and should aid surgeons in considering the use of RLN IONM in non-thyroid and non-parathyroid surgeries, thus potentially decreasing the rate of RLN injury in these procedures.

## 5. Conclusions

RLN IONM in non-thyroid/non-parathyroid surgery, although frequently used, has not been the subject of thorough reports, and only case reports or small retrospective series are available concerning tracheal resections and cervical oesophageal resection, but with no high-level evidence in any case. Nevertheless, in view of the close proximity of the RLN in surgical approaches to neoplasms and other diseases of the cervico-mediastinal junction area, trachea, and oesophagus such as in the excision of thoracic inlet neoplasms and other pathologies, using IONM self-evidently may potentially help in minimising unexpected RLN injury.

## Figures and Tables

**Table 1:** Studies found using the terms “oesophageal surgery” AND “recurrent laryngeal nerve monitoring”; “esophageal surgery” AND “recurrent laryngeal nerve monitoring”.

Author	Type of Article	Outcome
Hemmergling 2001 [41]	Case report	The use of surface laryngeal electrode for IONM in single-lung ventilation for oesophagectomy.
Gelpke 2010 [40]	Case series	IONM is feasible, easy, and reliable. It may reduce the incidence of permanent RLN paralysis.
Garas 2013 [15]	Review article	Supports the use of IONM during high mediastinal lymph node dissection in three-stage oesophagectomy.
Zhong 2014 [31]	Case series	CIONM is safe and effectively identifies the RLN. It may be a helpful method for decreasing the incidence of RLN paralysis.
Tsang 2016 [32]	Case report	CIONM is feasible during VATS oesophagectomy and can alert the surgeon of imminent injury to the RLN, thereby preventing permanent injury.
Wong 2017 [30]	Review article	CIONM has the potential to aid RLN dissection.
Hikage 2017 [39]	Case series	Confirms the feasibility and safety of IONM of the RLN for thoracoscopic oesophagectomy in the prone position.
Kobayashi 2018 [47]	Case series	The combination of IONM and the concept of the mesoesophagus has substantial advantages in allowing accurate and safe mediastinal lymphadenectomy during prone oesophagectomy.
Yuda 2018 [50]	Case series	Using IONM systematically, the prediction of RLNP and detection of nerve injury points seem feasible
Kitagawa 2019 [29]	Case report	Authors recommend using IONM to avoid injury to the non-recurrent inferior laryngeal nerve associated with the aberrant right subclavian artery.
Kanemura 2019 [46]	Case series	The absolute EMG amplitude of IONM might be helpful to predict the occurrence and severity of RLN palsy after oesophageal surgery.
Mushiake 2020 [28]	Case report	IONM may be useful for evaluation of the function of the RLN.
Yip 2020 [45]	Review article	IONM has contributed to a lower rate of recurrent laryngeal nerve palsy.
Staubitz 2020 [48]	Case series	RLN damage and subsequent postoperative vocal cord paresis can potentially be prevented by IONM.
Nitta 2020 [14]	Case report	Combined intraoperative identification and monitoring of RLN is a reasonable method but has been superseded by NIM.
Ninomiya 2020 [51]	Case report	IONM is useful in oesophageal cancer with a right aortic arch undergoing surgery.
Wang 2021 [42]	Systematic review	IONM is a feasible and effective approach to minimise RLNP.
Wong 2021 [43]	Retrospective review	CNM helped improve bilateral RLN lymphadenectomy.
Takeda 2021 [49]	Case series	IONM is useful in identifying RLN and may aid in reducing the incidence of RLN injury during oesophageal surgery.
Zhao 2022 [33]	Case series	Abnormal IONM signals can provide an accurate prediction of postoperative VCP incidence.
Yuda 2022 [34]	Case series	IONM helps to reduce the risk of postoperative RLNP after oesophageal cancer surgery.
Huang 2022 [35]	Case series	IONM could reduce the incidence of postoperative vocal cord palsy and pneumonia.
Nadkarni 2022 [36]	Case report	Technique description and IONM.
Komatsu 2022 [37]	Case series	CIONM for RLN contributes to a remarkable reduction in the risk of postoperative RLNP.
Lee 2024 [44]	Case series	IONM is a useful tool for reducing RLNP incidence and postoperative pneumonia after thoracoscopic surgery for oesophageal cancer.

IONM: intraoperative nerve monitoring. CIONM: continuous intraoperative nerve monitoring. RLN: recurrent laryngeal nerve. RLNP: recurrent laryngeal nerve palsy.

## Data Availability

Data sharing not applicable.
